# Differential Classical Conditioning of the Nocebo Effect: Increasing Heat-Pain Perception without Verbal Suggestions

**DOI:** 10.3389/fpsyg.2017.02163

**Published:** 2017-12-13

**Authors:** Anne-Kathrin Bräscher, Dieter Kleinböhl, Rupert Hölzl, Susanne Becker

**Affiliations:** ^1^Otto-Selz-Institute of Applied Psychology, Mannheim Centre for Work and Health, University of Mannheim, Mannheim, Germany; ^2^Department for Clinical Psychology, Psychotherapy, and Experimental Psychopathology, Johannes Gutenberg University of Mainz, Mainz, Germany; ^3^Department of Cognitive and Clinical Neuroscience, Central Institute of Mental Health, Medical Faculty Mannheim, Heidelberg University, Heidelberg, Germany; ^4^Alan Edwards Centre for Research on Pain, Faculty of Dentistry, McGill University, Montreal, QC, Canada

**Keywords:** nocebo effect, classical conditioning, implicit learning, behavioral psychology, awareness, heat-pain

## Abstract

**Background:** Nocebo effects, including nocebo hyperalgesia, are a common phenomenon in clinical routine with manifold negative consequences. Both explicit expectations and learning by conditioning are known to induce nocebo effects, but the specific role of conditioning remains unclear, because conditioning is rarely implemented independent of verbal suggestions. Further, although pain is a multidimensional phenomenon, nocebo effects are usually assessed in subjective ratings only, neglecting, e.g., behavioral aspects. The aim of this study was to test whether nocebo hyperalgesia can be learned by conditioning without explicit expectations, to assess nocebo effects in different response channels, and to exploratively assess, whether contingency awareness is a necessary condition for conditioned nocebo hyperalgesia.

**Methods:** Twenty-one healthy volunteers were classically conditioned using painful and non-painful heat stimuli that followed two different cues. The conditioned nocebo effect was assessed by subjective ratings of perceived stimulation intensity on a visual analog scale and a behavioral discrimination task, assessing sensitization and habituation in response to the same stimulation following the two cues.

**Results:** Results show a conditioned nocebo effect indicated by the subjective intensity ratings. Conditioned effects were also seen in the behavioral responses, but paradoxically, behavioral responses indicated decreased perception after conditioning, but only for subjects successfully conditioned as indicated by the subjective ratings. Explorative analyses suggested that awareness of the contingencies and the different cues was not necessary for successful conditioning.

**Conclusion:** Nocebo effects can be learned without inducing additional explicit expectations. The dissociation between the two response channels, possibly representing the conditioned and a compensatory response, highlights the importance of considering different outcomes in nocebo responses to fully understand underlying mechanisms. The present results challenge the role of explicit expectations in conditioned nocebo effects and are relevant with implications in clinical contexts, e.g., when transient adverse effects become conditioned.

## Introduction

Ranging from slightly increased pain during venipuncture, to the experience of side effects, to the development of life-threatening conditions (Cannon, 1942), nocebo effects occur in numerous contexts. Particularly in clinical practice and research they are crucial, worsening symptoms and corrupting tests of therapeutic approaches (Barsky et al., 2002). Well-known is nocebo hyperalgesia, a robust phenomenon often investigated in experimental and clinical contexts. A model derived from a series of studies (Benedetti et al., 2003) describes nocebo (and placebo) effects in pain as a process necessarily mediated by conscious expectations, i.e., expectations that a person can report (Stewart-Williams and Podd, 2004). Such expectations can be induced by instructional learning or verbal suggestion, but also by learning through classical conditioning (Kirsch, 2004) and observational learning (Vögtle et al., 2013). However, the potentially differential mechanisms of nocebo effects induced by conditioning or verbal suggestion have not been deciphered precisely in most previous studies. Usually, it is not directly tested whether nocebo hyperalgesia can be learned without explicit expectations. Typically, verbal suggestion alone is contrasted to a conditioning procedure plus verbal suggestion (e.g., Colloca and Benedetti, 2006; Colloca et al., 2008; Bingel et al., 2011; Reicherts et al., 2016) and/or a medical carrier substance (e.g., pill or cream) is used as the placebo/nocebo (Voudouris et al., 1985, 1989; van Laarhoven et al., 2011), which induces expectations from the outset because of participants’ earlier (unrelated) experience with such a substance (Barsky et al., 2002). Only few studies investigated nocebo effects when no classical nocebo such as a pill or a cream or a procedure like sham acupuncture is applied, but using verbal suggestion as the sole manipulation (Staats et al., 1998; Arntz and Claassens, 2004). Even fewer studies used classical conditioning without verbal suggestions and medically connoted nocebos to induce nocebo hyperalgesia (Jensen et al., 2012, 2014; Egorova et al., 2015, 2017). However, the research focus of these latter studies was on the question whether placebo and nocebo effects can be elicited by subliminally presented cues. It has been subject of debate for a long time whether expectations, especially induced by conditioning, exist only on a conscious level or whether unconscious expectations are effective as well (Stewart-Williams and Podd, 2004; Colloca and Miller, 2011). Accumulating evidence indicates that conditioned nocebo hyperalgesia does not necessarily depend on conscious processes, challenging the model by Benedetti et al. (2003). Particularly, results on successful induction of nocebo hyperalgesia with subliminally presented cues after learning of the response contradict the assumption that nocebo hyperalgesia has to be mediated by explicit expectations (Jensen et al., 2012, 2014; Egorova et al., 2015, 2017). Moreover, some results suggest that learning of nocebo hyperalgesia can occur with subliminal cues during the acquisition of the learning, although results for this specific condition were not reported separately (Jensen et al., 2015).

Because of their considerable effects nocebos can have in clinical practice and research, it is important to investigate the mechanisms at work. Learned nocebo responses without conscious expectations, for instance, cannot be directly assessed and counteracted (e.g., by the attending therapist) and therefore potentially induce hidden effects, like deteriorating therapeutic outcomes. Such hidden effects could also occur because conditioning can affect two or more dimensions of an experience differentially. Differential effects of conditioning have been shown, e.g., in verbal and behavioral pain responses, leading to a dissociation of these response channels (Becker et al., 2008) or verbal responses and measures of physiological processes, reflected in changes of cortisol levels (Johansen et al., 2003). Such differential responding might have important clinical implications leading for example to (unconscious or unnoticed) behavioral responses to pain, like exaggerated and/or persistent relieving postures that can contribute to chronic pain (Fordyce, 1976; Flor et al., 1990). Nocebo hyperalgesia has been investigated primarily in subjective pain reports. While being an important assessment of pain, subjective reports are prone to response biases (Kienle and Kiene, 1997; Hróbjartsson and Gøtzsche, 2001) and it is conceivable that subjective nocebo effects are caused (partly) by changes in response criteria (Cowey, 2004).

The aim of this study was (1) to investigate conditioning of nocebo hyperalgesia without explicit expectations, (2) to explore differential effects on different response channels by assessing heat-pain perception via subjective ratings and a behavioral discrimination task (Hölzl et al., 2005; Becker et al., 2008, 2011), and (3) to address the role of unconscious expectations by assessing contingency awareness in an explorative analysis. We hypothesized that conditioning without additional verbal suggestions would increase the perception of heat-pain stimuli indicated by increased subjective ratings of the intensity and increased behaviorally assessed sensitization.

## Materials and Methods

### Participants

Twenty-six healthy volunteers (12 females, age: *M* = 24.1 years, *SD* = 4.2) were recruited via mailing lists and participated after screening for the exclusion criteria. Exclusion criteria were chronic (longer than 3 months or more than once a month for longer than 3 days) or current acute pain, intake of pain medication or psychotropics, diabetes, hypertension, cardiopathy, thyroid disease, renal insufficiency, hepatic dysfunction, epilepsy, stroke, Parkinson’s disease, multiple sclerosis, psychiatric or neurologic diagnoses, intake of illegal drugs, alcohol, medication, or drug abuse, pregnancy, and left-handedness (tested with the Edinburgh Handedness Inventory; Oldfield, 1971). The sample size was determined by an *a priori* sample size calculation, assuming a medium effect size (*f* = 0.25) with a 5% probability for committing a Type I error (α = 0.05), a 20% probability for a Type II error (β = 0.80), an ANOVA design with two within-subject factors, and an attrition rate of 15%, resulting in 26 needed participants.

The experimental protocol was conducted in accordance with the Declaration of Helsinki and approved by the Local Ethics Committee. All participants gave written informed consent prior to experimental testing and received a monetary compensation of 30 € for participation.

### Conditioning Task

Classical conditioning is an associative learning paradigm, in which a neutral stimulus is paired with an unconditioned stimulus, i.e., a stimulus that elicits an unconditioned response. After repeated pairing, this neutral stimulus becomes a conditioned stimulus, i.e., it acquires the capacity to elicit a response similar to the unconditioned response, the conditioned response (cf. Stewart-Williams and Podd, 2004).

Participants took part in one experimental session of approximately 45 min duration, during which a conditioning task was performed after familiarization with the stimuli and scales used. One female experimenter (AKB) conducted all experiments and informed the participants about the experimental tasks and answered any questions before the start of the experiment. Participants were told that the experiment served the purpose to examine the perception of temperature and pain stimuli. They were not informed about the contingencies between cue and pain stimulus in order to allow conditioning without contingency awareness.

**FIGURE 1:**
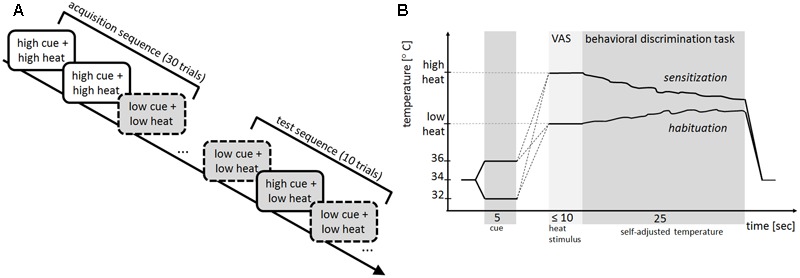
**(A)** Experimental procedure. In the acquisition sequence, 15 trials with the high cue (solid line) always preceded the high heat stimulus (white filling) and 15 trials with the low cue (dashed line) always preceded the low heat stimulus (gray filling). In the subsequent test sequence, both the high and low cue (five trials each) preceded the low heat stimulus. The sequence of cues was pseudorandomized across the acquisition and test sequence. **(B)** Trial structure of the conditioning task. Starting at baseline, the thermal stimulation increased or decreased to the intensity of the high or low cue (5 s), followed by an increase to the intensity of the high or low heat stimulus. Afterwards participants rated the perceived stimulus intensity on a visual analog scale (VAS). After this rating, the behavioral discrimination task started and participants kept the perceived stimulus intensity constant for 25 s (self-adjusted temperature); an example of sensitization after high heat (down-regulation of the temperature) and an example of habituation after low heat (up-regulation of the temperature) is shown. After this self-adjustment, the stimulation intensity returned to baseline temperature before the next trial started.

The conditioning task was divided into an acquisition and test sequence (**Figure 1A**). In the acquisition sequence, two cues were used. One cue contingently preceded a non-painful and other cue a painful heat stimulus. Each cue-heat combination was presented in 15 trials during acquisition. In the test sequence, both cues were followed by the non-painful heat in five trials each.

The trial order was pseudo-randomized for the acquisition and the test sequence with the constraint of no more than three subsequent presentations of the same cue.

### Outcome Measures

The conditioning task comprised two different assessments methods of perception. Intensity ratings on a visual analog scale (VAS) served as an explicit evaluation of perceived stimulus intensity at a given point in time. A vertically oriented VAS was labeled with 0 ‘warm’ at the bottom and 100 ‘very strong pain’ at the top, with an additional anchor at 40, labeled ‘just painful’ (Lautenbacher et al., 1992). An increased perceived intensity of the heat stimulus after the high cue compared to the low cue during the test sequence was considered a nocebo effect.

The second outcome measure was a behavioral response indicating perceived changes in pain perception in response to an ongoing stimulation. For this purpose, participants performed a previously established and validated behavioral discrimination task (Becker et al., 2008, 2011), in which participants were instructed to keep the perceived temperature constant by antagonizing any perceived change with a response unit (turning the wheel of a computer mouse up or down). Because the temperature did in fact not change other than when the participant operated the response unit, any change perceived by the participant due to habituation or sensitization was indicated by up- or down-regulation of the temperature. The behavioral response was calculated by subtracting the self-adjusted temperature at the end of the behavioral discrimination task from the temperature at the beginning. Increased sensitization in response to trials with high cue compared to low cue in the test phase was considered a nocebo effect.

### Time Course of a Conditioning Trial

Each trial of the conditioning task (**Figure 1B**) started with the presentation of one of two different thermal stimuli, namely 32°C vs. 36°C, as cues (for details on the determination of the cues see section “Cues”), i.e., the stimulation decreased or increased from a baseline temperature of 34°C to 32°C or 36°C. After 5 s at the temperature of the respective cue, the temperature increased until it reached the designated temperature of either the high or low heat stimulus, employing a trace conditioning design with minimal delay. When this target temperature was reached, participants rated the perceived intensity of the stimulus on the VAS. After this rating, participants performed the behavioral discrimination task. After 25 s, the temperature returned to baseline and the next trial started after a short break (5–10 s).

Participants received the following instruction (in German): “After an initial change in temperature, the temperature increases and you will by prompted by a message on the screen to rate your current sensation at your thenar eminence by means of the familiar rating scale. After that you will be asked to keep the temperature constant. That means that you should countersteer every change in temperature that you feel with the help of the computer mouse so that the initial temperature will be retained. You will be informed about the end of the temperature adjustment on the screen. After that the trial ends and the temperature decreases. After a short break, the next trial begins.”

### Cues

As mentioned above, two different thermal stimuli (32°C or 36°C) were used as cues (1) to account for aspects of natural relations (with the cue being a precursor of the subsequent heat stimulus) instead of coupling of arbitrary stimuli (e.g., colored squares or circles) and (2) to reduce cross-modal traffic (all stimuli were applied within the same somatosensory afferent system) in order to facilitate conditioning (c.f. Rescorla and Furrow, 1977; Cusato and Domjan, 2012). The cue temperatures and baseline temperature were fixed and identical for every participant. The baseline temperature of 34°C was chosen because this lies well within the neutral or indifference zone (30–36°C) leading to a neutral sensation of neither warm nor cold. Further, choosing cue temperatures of 32°C and 36°C led to a sensation of ‘warm’ or ‘cold’ within the neutral zone (i.e., if held constant for a few minutes would have led to a neutral sensation again). These temperatures were chosen to use maximally and equally neutral cues and optimized in a pilot test. The stimulus intensities of both cues were below the pain threshold for all participants. Pairing of the two cues with the two heat stimuli was balanced across participants, i.e., in half of the participants the 32°C cue and in the other half the 36°C was coupled to the high heat stimulus.

### Heat Stimuli

The intensities of the high and low heat stimuli were adjusted to participants’ individual pain thresholds assessed prior to the conditioning task with the method of adjustment (Kleinböhl et al., 1999). For this threshold assessment, participants increased the stimulation temperature themselves with the response unit, starting from baseline (34°C) until they perceived the temperature as just painful. Then the temperature returned to baseline. This assessment was repeated 3–6 times (taking into account inter-trial habituation processes) until a robust temperature representing the pain threshold was reached. The just painful self-adjusted temperature of the last trial was employed as the pain threshold (Kleinböhl et al., 1999). Intensities of the high and low heat stimuli were this pain threshold plus/minus four units of just noticeable differences in the painful and non-painful range (Bushnell et al., 1985; Maixner et al., 1986, 1989), resulting in pain threshold + 1.5°C for the high heat and pain threshold -2.2°C for the low heat stimulus.

### Presentation of Stimuli

All thermal stimuli were applied with a contact heat thermode (SENSELab-MSA Thermotest, SOMEDIC Sales AB, Sweden) with a size of 25 mm × 50 mm. This thermode system allows for phasic and tonic stimulation within a temperature range from 10 to 52°C with a relative accuracy of 0.02°C. The rate of temperature change, i.e., time in which the temperature changed from baseline to cue temperature or from cue temperature to the level of the heat stimulus, was 0.7°C/s, except at the end of a trial where the temperature returned to baseline with a rate of 3°C/s. The thermal stimuli were presented at the thenar eminence of the participants’ left hand. To prevent skin damage, the maximum temperature was limited to 50°C and total applied energy was restricted by integrating temperature over time. The procedure was terminated if a critical value was reached. This value was calculated according to human and animal data on skin burns through contact heat (Lamotte, 1979; Dahl et al., 1993; Brennum et al., 1994; Pedersen et al., 1998). The experimental procedures were automatized and controlled by a separate personal computer coupled to the thermostimulator system. A computer screen in front of the participant displayed short instructions (i.e., “Rate the intensity,” “Keep the temperature constant”) during task performance to remind participants of the specific subtasks of each trial interval and the rating scales. A computer mouse with two buttons and a wheel served as response unit.

### Post-experimental Interview and Questionnaires

In order to assess awareness of the different cues and the contingencies, participants were interviewed at the end of the test session. For this purpose, they were shown a flowchart depicting one trial of the conditioning task, divided into the sections “first temperature change” (cues), “second temperature change” (heat stimuli), “temperature rating” (VAS rating), and “temperature retention interval” (behavioral discrimination task). To assess if the participants discriminated the cues, they were asked if they had felt different intensities in different trials during the “first temperature change” (yes/no question). To assess contingency awareness, they were asked if they had been able to predict the “second temperature change” (yes/no question). In case of affirmation, we asked how they were able to predict the “second temperature change” (open question). In case of negation, we inquired if there could have been any relation between the first and second temperature change (open question).

At the end of the testing session, participants also filled in both the state and trait part of the State-Trait Anxiety Inventory (STAI; Spielberger et al., 1970) and the Fear of Pain Questionnaire (McNeil and Rainwater, 1998) because previous studies found an association of nocebo hyperalgesia and anxiety measures (Colloca et al., 2010; Bingel et al., 2011).

### Statistical Analysis

Five participants were excluded from further statistical analyses because they rated the high heat as non-painful (i.e., <40 on the VAS on average) during the acquisition sequence, resulting in 21 participants in the statistical analyses. To assess nocebo effects, only responses during the test sequence were considered.

#### Analyses of the Nocebo Effect Measured by Perceived Intensity Rating

After confirming the normal distribution of the residuals, a linear mixed model for an ANOVA design with repeated measurements with two within-subjects factors, ‘cues’ (high vs. low) and ‘trial’ (5 trials) and the subjective rating as dependent variable was used to assess the effects of the conditioning task. In two further linear mixed models we assessed whether the coupling and gender, respectively, influenced the conditioning effect. We used the same factors and dependent variable and an additional between-subjects factor ‘coupling’ (32°C or 36°C coupled to the painful heat stimulus) or ‘gender’ (female vs. male). Further, linear mixed models with the same factors and additional covariates were calculated to rule out alternative explanations for differences in VAS ratings in the test sequence. Covariates were the pain threshold and extent of sensitization or habituation (i.e., slopes of a regression, in which trial was used to predicted the VAS rating of the high stimulus during the acquisition) used in different models.

After testing for multivariate outliers with Mahalanobis distance, Pearson’s correlations were calculated to assess the relationship between nocebo effects in the test sequence and VAS ratings of the heat stimuli in the acquisition sequence (*N* = 20 due to one bivariate outlier).

#### Analyses of the Nocebo Effect in Behavioral Discrimination

As before, linear mixed models for an ANOVA design with repeated measurements with two within-subject factors, ‘cue’ and ‘trial,’ were used to assess the effects of the conditioning task on the behavioral response as dependent variable. With one sample *t*-tests testing against zero, it was tested whether the behavioral responses for the high and low cues during acquisition and test sequence, respectively, could be identified as sensitization (values below 0) or habituation (values above 0).

*Post hoc*, participants were divided according to their VAS ratings into successfully conditioned participants (‘responders’) and participants, who showed no conditioned response (‘non-responders’). Participants were considered responders, if they rated the high cue on average higher than the low cue in the test phase. This categorization was solely based on VAS ratings and only used for further independent analyses of the behavioral responses avoiding circular reasoning (Kriegeskorte et al., 2009). To test whether the subgroups differed in their behavioral responses, a linear mixed model with the between-subjects factor ‘subgroup’ (responders vs. non-responders) and the within-subjects factor ‘cue’ was employed, followed by *post hoc* tests (Fisher’s least significant differences; LSD) where appropriate.

#### Analyses of Responder/Non-responder Characteristics

Spearman’s correlations (due to non-normally distributed variables) were calculated assessing the relationship between the difference in VAS ratings of trials cued with the high and the low cue in the acquisition contrasted to the test phase in the responder and non-responder subgroups. Differences in responders and non-responders with regard to the VAS ratings in response to the high and low cue during acquisition and test sequence, respectively, were tested with Mann–Whitney *U* tests, due to deviations from the normal distribution. *t*-tests were calculated to test whether pain thresholds differed between responders and non-responders.

#### Further Correlational Analyses

Pearson’s correlations were calculated to assess the relationship between the subjective and behavioral assessments, and between subjective and behavioral nocebo effect and measures of anxiety.

#### Analyses of Contingency Awareness

The necessity of contingency awareness and of awareness of the two different cues in the conditioned nocebo effect was tested by a previously described regression method (Greenwald et al., 1995; Becker et al., 2012), by which awareness and cue differentiation predicted the nocebo effect. The intercept of this regression (level of the regression line) estimates the size of the nocebo response without awareness and cue differentiation, respectively, e.g., an intercept larger than zero means that a nocebo effect occurs independent of the awareness. The slope of this regression (steepness of the regression line) indicates whether awareness or cue differentiation, respectively, is beneficial for the development of a nocebo effect, e.g., a slope larger than zero means that there is a positive relation between awareness and the nocebo effect.

For all analyses, effect sizes were calculated and interpreted due to the limited sample size. For all linear mixed models, we estimated generalized η^2^ as an effect size (Olejnik and Algina, 2003) and interpreted values of 0.02 as small, 0.13 as medium, and 0.26 as large (Bakeman, 2005). The significance level was set to 5%. Analyses were calculated in SPSS 22.

## Results

### Subjective Ratings in the Acquisition Sequence

**FIGURE 2:**
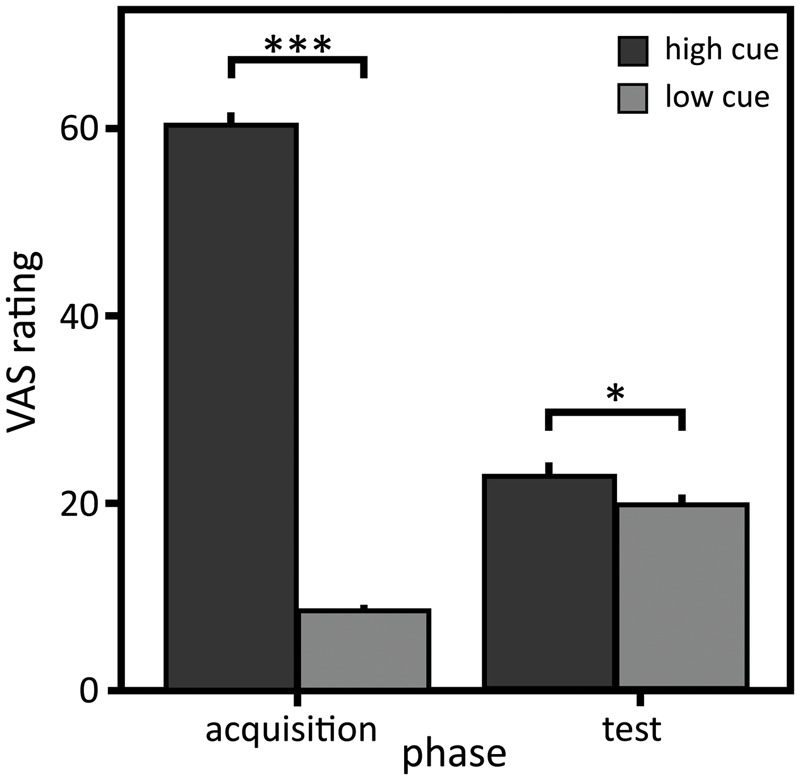
Visual analog scale (VAS) ratings of the perceived intensity. Mean and standard errors of mean of the VAS ratings for the high and low cue in the acquisition and the test sequence (*N* = 21). ^∗^*p* < 0.05; ^∗∗∗^*p* < 0.001.

Presentation of the low heat (*M* = 41.2°C, *SD* = 2.64) and the high heat stimulus (*M* = 44.9°C, *SD* = 2.64) resulted in a mean rating of 8.5 (*SD* = 9.17) for the low and 60.8 (*SD* = 15.72) for the high heat stimulus on the VAS in the acquisition sequence (see **Figure 2**). Thus, as intended, the low heat stimulus was perceived as non-painful, while the high heat stimulus was perceived as painful.

### Nocebo Effect Measured by Perceived Pain Intensity

Despite identical physical stimulation intensities, VAS ratings in response to the warm cue coupled to the high heat during acquisition (‘high cue’) compared to the warm cue coupled to the low heat during acquisition (‘low cue’) were significantly higher in the test sequence, indicating a small to medium conditioned nocebo effect [**Tables 1, 2**; main effect of ‘cue’: *F*(1,40) = 5.37, *p* = 0.026, η*^2^* = 0.07]. Importantly, the different coupling (32°C or 36°C as high cue) neither influenced the VAS rating [main effect of ‘coupling’: *F*(1,21) = 2.35, *p* = 0.140, η^2^ = 0.11] nor the conditioning effect [interaction effect ‘coupling’ × ‘cue’: *F*(1,40) = 0.83, *p* = 0.368, η^2^ = 0.02; main effect of ‘cue’: *F*(1, 40) = 6.43, *p* = 0.015, η^2^ = 0.17]. The intensity rating differed with respect to gender [females: *M* = 9.91, *SD* = 9.27; males: *M* = 30.17, *SD* = 21.58; main effect ‘gender’: *F*(1,91) = 27.07, *p* < 0.001, η^2^ = 0.51] but the interaction between gender and cue was not significant [interaction effect ‘gender’ × ‘cue’: *F*(1,128) = 3.43, *p* = 0.066, η^2^ < 0.01], leading to the assumption that gender did not influence the nocebo effect observed here.

**Table 1:** Descriptive results on the subjective rating of intensity (visual analog scale, VAS) and the behavioral discrimination.

Outcome measure	Experimental phase	High cue trials *M (SD)*	Low cue trials *M (SD)*
VAS	Acquisition	60.46 (21.09)	8.51 (11.26)
	Test	22.92 (22.15)	19.89 (17.64)
Behavioral discrimination	Acquisition	-0.13 (0.33)	-0.01 (0.46)
	Test	0.16 (0.34)	0.10 (0.37)
	Test (responders)	0.23 (0.32)	0.10 (0.36)
	Test (non-responders)	0.04 (0.35)	0.10 (0.40)

**Table 2:** Repeated measures analysis of subjective and behavioral measures of the nocebo effect.

Effect	Outcome measure *F* (df num; df den); *p*^a^
	*Subjective rating (VAS)*
*Main effects^b^*	
Cue (high, low)	5.37(1; 40); 0.026
Trial [1–5]	4.64 (4; 86); 0.002
*Interaction effect*	
Cue × Trial	1.04 (4; 95); 0.393
	*Behavioral discrimination*
*Main effects^c^*	
Cue (high, low)	1.42 (1; 36); 0.242
Trial [1–5]	1.20 (4; 104); 0.316
*Interaction effect*	
Cue × Trial	0.07 (4; 89); 0.991
	*Behavioral discrimination, with non-/responder subgroups*
*Main effects^d^*	
Cue (high, low)	0.65 (1; 188); 0.421
Subgroup (non-/responders)	1.35 (1; 21); 0.258
*Interaction effect*	
Cue × Subgroup	4.38 (1; 188); 0.038

*Results of analyses with linear mixed models for an ANOVA design with repeated measures ^b^for the subjective rating (VAS, visual analog scale), ^c^for behavioral discrimination, and ^d^dividing the sample into subgroups of responders and non-responders, during the test phase. ^a^Adjusted F-ratios, degrees of freedom for denominators (den) and for numerators (num) in brackets and exact probabilities for main effects and interactions.*

Perceived intensity of the heat stimuli preceded by either cue during the acquisition positively correlated with the nocebo effect (high cue *r* = 0.63, *p* = 0.002; low cue *r* = 0.51, *p* = 0.022), suggesting that participants perceiving the stimulation as more intense developed a larger conditioned nocebo effect. Differences in pain thresholds, determining intensities of the heat stimuli [main effect of the covariate ‘pain threshold’: *F*(1,91) = 18.4, *p* < 0.001, η^2^ = 0.16; interaction effect ‘cue’ × ‘pain threshold’: *F*(1,129) = 0.46, *p* = 0.497, η^2^ = 0.01] or sensitization or habituation across trials across high stimulus trials during acquisition [main effect of the covariate ‘perceptual change’: *F*(1,92) = 14.14, *p* < 0.001, η^2^ = 0.17, interaction effect ‘perceptual change’ × ‘cue’: *F*(1,129) = 0.01, *p* = 0.923, η*^2^* = 0.01] could not explain the nocebo effect as it did not covary with either variable.

### Nocebo Effect in Behavioral Discrimination

**FIGURE 3:**
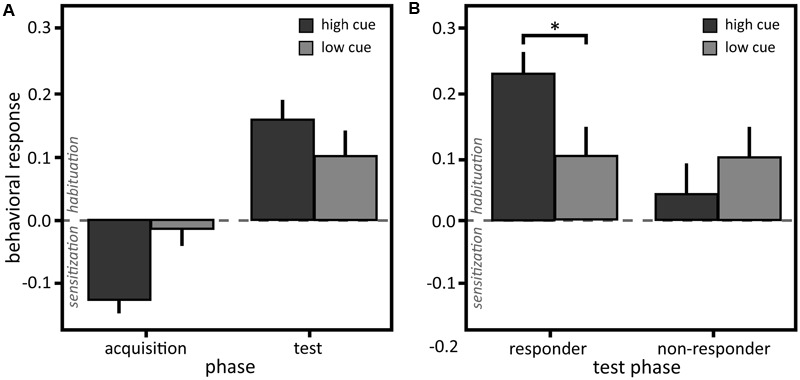
**(A)** Behavioral responses to the stimulation. Mean and standard errors of mean of the difference between self-adjusted temperature and initial temperature of the behavioral discrimination task for the high and low cue in the learning and test sequence of the whole sample (*N* = 21) and **(B)** of the responder (*N* = 13) and non-responder subgroups (*N* = 8) in the test sequence. ^∗^*p* < 0.05.

Despite a small effect, the behavioral responses were not significantly different between the low and the high cue in the test sequence [**Tables 1, 2**; main effect ‘cue’: *F*(1,36) = 1.42, *p* = 0.242, η^2^ = 0.02]. The following tests were conducted using Bonferroni adjusted alpha levels of 0.0125 per test (0.05/4). Implying a dissociation from the results of the subjective ratings, participants habituated in response to the stimulation after the high cue in the test phase [*M* = 0.21, *SD* = 0.217, *t*(20) = 4.41, *p* < 0.001, Cohen’s *d* = 0.96]. This result contradicts the subjective intensity ratings that indicate increased sensation after the high cue. After the low cue, a small effect size suggests some habituation in response to the stimulation, although the mean is close to zero [*M* = 0.05, *SD* = 0.198, *t*(20) = 1.22, *p* = 0.235, Cohen’s *d* = 0.27] (see **Figure 3A**). In the acquisition, participants sensitized [*M* = -0.12, *SD* = 0.194, *t*(20) = -2.87, *p* = 0.009, Cohen’s *d* = 0.63] in response to the high heat and neither habituated nor sensitized [*M* = -0.01, *SD* = 0.233, *t*(20) = -0.26, *p* = 0.801, Cohen’s *d* = 0.06] in response to the low heat (see **Figure 3A**), as expected (c.f. Kleinböhl et al., 1999).

Dividing the sample, participants who were successfully conditioned showing a nocebo effect in their VAS ratings (‘responders’; 13 participants, 62%) habituated significantly more in response to the high cue, displayed in their behavioral assessment, compared to non-responders [see **Figure 3B** and **Tables 1, 2**; interaction ‘cue’ × ‘non-/responders’: *F*(1,188) = 4.38, *p* = 0.038, η^2^ = 0.20; *post hoc* comparison responders: high vs. low cue *LSD* = 0.13, *p* = 0.020, Cohen’s *d* = 0.91].

### Characteristics of the Responder and Non-responder Subgroups

For the responders only, the difference in perception of the high and low stimuli in the acquisition sequence correlated positively with this difference in the test sequence (responders: *r*_s_ = 0.67, *p* = 0.013, non-responders: *r*_s_ = -0.10, *p* = 0.806). This shows that the bigger participants perceived the difference between the high and low stimuli in the acquisition, the bigger was the nocebo effect in the test phase, indicating that the magnitude of the nocebo effect was associated with subjective pain perception during the acquisition. Further, responders perceived the low heat in the acquisition phase as more intense compared to the non-responders (responders *M* = 11.2, *SD* = 10.77, non-responders *M* = 4.1, *SD* = 2.31, *U* = 84.5, *p* = 0.016, Cohen’s *d* = 0.91). A medium effect size suggests that responders also perceived the high heat as more intense compared to the non-responders, although this difference did not reach statistical significance (responders *M* = 64.5, *SD* = 17.69, non-responders *M* = 54.8, *SD* = 10.15, *U* = 69, *p* = 0.238, Cohen’s *d* = 0.67). Similarly, in the test phase responders perceived the low heat (preceded by both the low and the high cue) as more intense than the non-responders (low cue, responders *M* = 25.5, *SD* = 19.04, non-responders *M* = 10.7, *SD* = 3.53, *U* = 80, *p* = 0.045, Cohen’s *d* = 1.08; high cue, responders *M* = 32.5, *SD* = 22.22, non-responders *M* = 7.5, *SD* = 4.09, *U* = 97, *p* < 0.001, Cohen’s *d* = 1.57). Pain thresholds and therefore stimulus intensities were not different between responders and non-responders, albeit a medium effect [pain threshold responders: *M* = 43.9°C, *SD* = 2.64; non-responders: *M* = 42.6°C, *SD* = 2.59; *t*(19) = -1.13, *p* = 0.274, Cohen’s *d* = 0.50], suggesting that subjective perception rather than stimulus intensity is relevant for the nocebo effect.

### Relation of the Subjective and Behavioral Assessment

**FIGURE 4:**
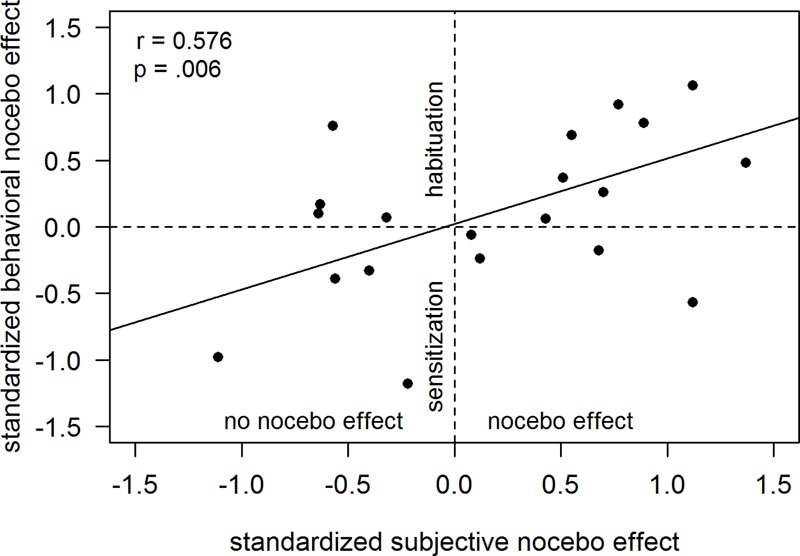
Correlation of the nocebo response in the subjective ratings and the behavioral responses. Nocebo responses in subjective ratings and behavioral responses are displayed as standardized differences between the high and low cue.

Across the whole sample, the larger the conditioned nocebo response in the VAS ratings, the larger was the habituation displayed in the behavioral response (see **Figure 4**): z-standardized nocebo responses in VAS ratings (calculated as the difference in response to trials cued with the high versus low cue) correlated positively with z-standardized nocebo responses in the behavioral assessment (*r* = 0.58, *p* = 0.006). No other correlations between responses in the subjective and behavioral assessment were found.

### Explorative Analysis of Contingency Awareness

Fourteen (67%) participants were unable to report the contingencies, i.e., which cue was coupled to which heat stimulus, after the experiment, but 9 out of these 14 (64%) unaware participants were responders. Out of the seven participants, who recognized the contingencies, only four were responders. In response to the open questions (“How could you predict the “second temperature change?” and “Was there any relation between the first and second temperature change?”), most participants could either clearly explain the relationship (i.e., “the painful temperature came after the cooler temperature”) or they had no idea about the relationship and thought that the different temperatures came completely at random. Some participants emphasized the contrast in sensation between the first and the second temperature change and we counted this as contingency aware if the description matched the according coupling of cue and heat stimulus. Predicting contingency awareness by the nocebo effect in the VAS ratings revealed that learning success was independent of contingency awareness, indicated by an non-zero intercept of 2.6 and a medium effect size [*t*(20) = 1.26, *p* = 0.221, Cohen’s *d* = 0.57] in the regression analysis. A small effect size suggested that developing contingency awareness promoted nocebo effects at most partially [slope = 1.6, *t*(20) = 0.46, *p* = 0.653, Cohen’s *d* = 0.20]. In sum, according to explorative analyses, contingency awareness appeared not to be a sufficient condition for successful conditioning.

### Explorative Analysis of Awareness of the Different Cues

Nine participants (43%) did not discriminate the two cues and none of them was aware of the contingency, but six (67%) out of these nine participants were responders. Out of the 12 aware participants, 7 (58%) were responders. Predicting awareness of the different cues, i.e., discriminating the two warm cues, by the nocebo effect in the VAS ratings suggests that successful conditioning was independent of this awareness [intercept = 3.61, *t*(20) = 1.41, *p* = 0.174, Cohen’s *d* = 0.63]. Awareness of the different cues did not promote conditioning [slope = -0.86, *t*(20) = -0.26, *p* = 0.801, Cohen’s *d* = 0.11]. In sum, according to explorative analyses, awareness of the different cues was not a sufficient condition for successful conditioning.

### The Role of Anxiety for the Conditioned Nocebo Effect

Previous studies described anxiety as a possible factor explaining interindividual variations in nocebo responses (Benedetti et al., 2006; Colloca et al., 2010; Bingel et al., 2011) and one study specifically found that fear of medical pain was associated with nocebo hyperalgesia (Aslaksen and Lyby, 2015). Here, although not significant, a medium effect for the correlation of the nocebo effect in the behavioral assessment with trait anxiety was found (*r* = 0.29, *p* = 0.206). No other correlations of nocebo effects in VAS ratings or the behavioral assessment with state or trait anxiety and fear of pain were found (all *r* < 0.18, all *p* > 0.465).

## Discussion

It has been suggested that conditioning is not an important mechanism in nocebo hyperalgesia (compared to placebo hypoalgesia; Colloca et al., 2008) and according to one often-cited model, conscious expectations are imperative for the development of nocebo pain responses (Benedetti et al., 2003; Stewart-Williams and Podd, 2004). Here we show that after a conditioning procedure without verbal suggestions or application of medically connoted cues such as pills or cream, avoiding explicit *a priori* expectations, the same heat stimulus was rated significantly higher after a cue paired before to a painful stimulation compared to a cue paired before to a non-painful stimulation. Thus, the present results highlight the importance of conditioning in nocebo hyperalgesia. Few studies investigated conditioning effects in nocebo hyperalgesia without verbal suggestions and medically connoted nocebos (Jensen et al., 2012, 2014; Egorova et al., 2015, 2017). Further, one study on itch using colored lights as cues found a nocebo effect after conditioning plus verbal suggestion, but not after conditioning only (Bartels et al., 2014). In contrast, a recent study that also used colored lights as cues found placebo hypoalgesia and nocebo hyperalgesia after conditioning without additional verbal suggestions (Babel et al., 2017). Interestingly, a nocebo effect could be induced in our study using thermal cues, in order to facilitate conditioning due to natural relations (the cue being a precursor of heat-pain) and by reducing cross-modal traffic (all stimuli were applied within the same afferent system; Rescorla and Furrow, 1977; Cusato and Domjan, 2012). Although the nocebo effect is a complex phenomenon comprising more than the effects investigated here, conditioned nocebo effects have clinically relevant implications. Known from conditioning literature, conditioned nocebo effects likely possess characteristics that distinguish them from expectancy-induced effects. For instance, latent inhibition, i.e., decremental effects of non-reinforced pre-exposure to the to-be-conditional stimulus on subsequent learning (Lubow, 1973), induced by yearlong experience of ineffective therapies, a typical experience of many chronic pain patients, is assumed to have a negative impact on later interventions (Voudouris et al., 1985; Klinger et al., 2007; Bingel et al., 2011). It is also conceivable, for example, that typical transient adverse effects in the beginning of a (pharmaceutical) treatment are being consolidated by conditioning and thereby lead to conditioned nocebo effects in form of sustained adverse effects.

Nocebo effects occurred only in the subjective response channel, i.e., the VAS ratings. In contrast to this increased perception, the behavioral assessment showed decreased perception, i.e., habituation to ongoing stimulation, demonstrating a dissociation between both response channels. Appearing paradox at a first glance, similar dissociations between subjectively and behaviorally assessed perception are known in the context of pain from previous studies using VAS ratings and behavioral responses of habituation and sensitization (Kleinböhl et al., 1999) and operant learning paradigms (Hölzl et al., 2005; Becker et al., 2011) and in other perceptual domains, e.g., blindsight (Weiskrantz, 2004). Similarly, subjective pain ratings in another nocebo study did not reflect a nocebo response indicated by increased physiological stress parameters, demonstrating a dissociation of subjective and physiological response channels (Johansen et al., 2003). These findings highlight that pain perception is multidimensional with the dimensions being at least partially independent. Thus, the behavioral assessment proved to be an important complementary assessment, capturing different, not necessarily verbally representable aspects of perception compared to subjective ratings. Further, as an objective method, it reduces the risk to confound changes in response criteria with changes in perception (Cowey, 2004) and is less jeopardized by demand characteristics (Kienle and Kiene, 1997; Hróbjartsson and Gøtzsche, 2001).

The apparent paradox of increased intensity ratings followed by increased behaviorally assessed habituation might be explained by different reference points (i.e., time point and dynamics) of the assessment methods: The bigger the nocebo effect in the subjective ratings, the larger the subsequent return of the sensory signal over time to a more “veridical” perception, better matching the physical stimulation. Further, this apparent paradox reminds of findings on conditioned opposing reactions to drugs in rodents, possibly explaining drug tolerance (Krank, 1987). Depending on the applied assessment method, conditioned reactions in the opposite direction than suggested by the drug were reported. Such effects are known as compensatory or antagonistic conditioned responses, diminishing unconditioned drug effects and leading to tolerance (Flaten et al., 1997; Domjan, 2005). Drug tolerance can emerge in physiological systems that are homeostatically regulated, i.e., that can show compensatory adjustments (Domjan, 2005). While drug effects have a conscious, reportable aspect, the compensatory response occurs implicitly, i.e., cannot be reported. This might explain that opposing reactions can be found depending on the assessment method. Considering pain a homeostatic emotion (Craig, 2003), the conditioned decreased perception in terms of habituation could represent an attempt of the nociceptive system to countersteer the conditioned increase in perception due to the nocebo effect. Similar to drug and compensatory effects, which response is observable might depend on the assessment method, i.e., whether an objective method assessing implicit processes or a subjective method assessing explicit processes is applied. This interpretation of the habituation as a compensatory response that is dependent on the reportable conditioned response represented in the subjective ratings is supported by the observed positive correlation, indicating that the conditioned behavioral scales with the conditioned subjective response.

A conditioned nocebo effect could be induced in 13 out of 21 participants, which is no exception in conditioning and placebo studies. Approximately only 33% of the participants in placebo studies typically show a placebo response (Hoffman et al., 2005). It is unknown whether similar responder rates apply to the nocebo effect because such rates are not reported in previous studies. While other studies on placebo effects typically used a median split and divided the sample into high and low responders (Scott et al., 2007, 2008; Elsenbruch et al., 2012), the criterion employed here was based on the differential response to the two cues in the VAS ratings, ensuring that only participants really showing a nocebo effect were categorized as responders. The responder criterion was supported by the positive correlation between perceived difference in stimulation intensity in the acquisition and the nocebo effect in the test phase that only occurred in the responder subgroup. Compared to reported responder rates in placebo studies, a rate of 62% nocebo responders in this study appears high, speaking in favor of a high effectiveness of nocebo conditioning, in contrast to earlier suggestions (Colloca et al., 2008). From a methodological point of view it is worth mentioning that the described responder criterion was applied only for further analyses of an independent variable, avoiding capitalization of chance (Kriegeskorte et al., 2009).

Several previous studies assessed the question whether nocebo responders possess specific characteristics compared to non-responders (Drici et al., 1995; Barsky et al., 2002; Vögtle et al., 2013; Webster et al., 2016, for review). Knowing whether a person will develop a nocebo effect would allow pre-selection of patients for special treatments or adapt the course of action in a clinical setting to reduce those effects. Some evidence points to anxiety as an important factor in this context (Colloca et al., 2010; Bingel et al., 2011), in line with the notion that anxiety-triggered cholecystokinin activation might cause nocebo hyperalgesia (Benedetti et al., 1997). Our results only partially support the role of anxiety, in that we found a small effect of trait but not state anxiety nor fear of pain. However, participants who perceived the stimulation as more intense were more prone to developing a nocebo effect and responders and non-responders differed in their pain sensitivity to some degree, suggesting that pain sensitivity might represent a general risk factor for developing a nocebo effect.

Explorative analyses of the present data suggest that some participants learned the nocebo effect without contingency awareness. Further, it seems unnecessary to consciously discriminate cues for successful conditioning of a nocebo effect, which is in line with a study showing that nocebo responses can be triggered by non-conscious cues (Jensen et al., 2012; Egorova et al., 2015). If confirmed in larger samples, these findings would have far reaching consequences, suggesting that patients might learn associations between various cues and subsequent pain increase without being able to recognize what caused the worsening. This prevents patients from developing pain control strategies and could lead to distrust in therapeutic efficacy, aggravation of illness, and thereby unnoticeably contribute to the maintenance of chronic pain (Flor et al., 1990). However, we only assessed contingency awareness, i.e., the conscious recognition of the CS-US relation and awareness of the different cues using a post-experimental interview. Post-experimental interviews may not be the optimal method of assessment and have been criticized (Labar and Disterhoft, 1998; Lovibond and Shanks, 2002), but have been used successfully (cf. Clark and Squire, 1998; Manns et al., 2001; Tabbert et al., 2006). In general, there has been a long-lasting debate on the possibility of conditioning in the absence of contingency awareness. In short, some belief that conditioning necessarily depends on contingency awareness (e.g., Dawson, 1973; Lovibond and Shanks, 2002; Mitchell et al., 2009; Lovibond et al., 2011). The main criticism relates to methodological issues, such as lacking sensitivity and specificity in assessing contingency awareness. Others are confident that implicit conditioning is possible based on theoretical considerations, which are, for example, supported by measures of brain activation (Clark et al., 2002; Wiens and Öhman, 2002; although one has to be very careful particularly with reverse inferences). Further support is seen in high quality studies comprising physiological (Benedetti et al., 2003) and autonomic responses (Manns et al., 2001; Knight et al., 2003, 2006), evaluative judgments (De Houwer et al., 1997, 2001), involuntary ventilation (Gallego and Perruchet, 1991) as well as other behavioral measures (Jensen et al., 2015), suggesting the occurrence of implicit conditioning.

Some limitations of the present study should be considered. Despite conducting an *a priori* sample size calculation, attrition rate was higher than expected and the resulting sample size was rather small, especially when it comes to subgroup analyses. We reported effect sizes to deal with this limitation. Further, the analyses on contingency awareness has only explorative character and should be replicated with larger samples and experimental designs that specifically test this aspect. Another restriction concerns the fact that the ratings in the test phase were on average in the subjectively non-painful range (i.e., <VAS 40). Although the applied temperatures (*M* = 41.2°C, *SD* = 2.64) evoke activation of nociceptive fibers (Torebjork et al., 1984; Treede et al., 1995, 1998), it can be discussed whether the results represent what is traditionally termed nocebo effect. In line with this thinking, a study using electric stimuli below the pain threshold demonstrated increased tactile sensations and enhanced somatosensory cortical responses after conditioning and verbal suggestion, refraining from labeling this a placebo or nocebo effect (Fiorio et al., 2012 also refer to Beissner et al., 2015).

In summary, this study provides experimental evidence that nocebo effect in heat-pain perception can be classically conditioned, demonstrating cognitive-emotional pain modulation without verbal suggestions. Although pain-facilitating effects were only found in the subjective response channel, the objective behavioral response channel suggests simultaneous pain-inhibition, possibly as a compensatory reaction. Future studies should investigate the precise mechanisms of the dissociation between different response channels and under which conditions behavioral responses show nocebo or compensatory effects, as well as replicate our findings indicating that contingency awareness is not a necessary condition for successful conditioning of nocebo hyperalgesia.

## Author Contributions

A-KB, SB, and RH designed the study; A-KB performed experiments and analyzed the data; A-KB, SB, and RH wrote the manuscript; DK gave technical support; DK and RH gave conceptual advice. All authors discussed the results and commented on the manuscript.

## Conflict of Interest Statement

The authors declare that the research was conducted in the absence of any commercial or financial relationships that could be construed as a potential conflict of interest.
